# Brain perfusion SPECT in dementia: what radiologists should know

**DOI:** 10.1007/s11604-024-01612-5

**Published:** 2024-06-18

**Authors:** Tomoki Imokawa, Kota Yokoyama, Kanae Takahashi, Jun Oyama, Junichi Tsuchiya, Nobuo Sanjo, Ukihide Tateishi

**Affiliations:** 1 Department of Diagnostic Radiology, Tokyo Medical and Dental University, Bunkyo-Ku, Tokyo, Japan; 2 Department of Radiology, Japanese Red Cross Omori Hospital, Ota-Ku, Tokyo, Japan; 3 Department of Neurology and Neurological Science, Graduate School of Medical and Dental Sciences, Tokyo Medical and Dental University, Bunkyo-Ku, Tokyo, Japan

**Keywords:** Brain perfusion single-photon emission computed tomography, Alzheimer’s disease, Dementia with Lewy bodies, Frontotemporal dementia, Idiopathic normal pressure hydrocephalus, Prion diseases

## Abstract

The findings of brain perfusion single-photon emission computed tomography (SPECT), which detects abnormalities often before changes manifest in morphological imaging, mainly reflect neurodegeneration and contribute to dementia evaluation. A major shift is about to occur in dementia practice to the approach of diagnosing based on biomarkers and treating with disease-modifying drugs. Accordingly, brain perfusion SPECT will be required to serve as a biomarker of neurodegeneration. Hypoperfusion in Alzheimer’s disease (AD) is typically seen in the posterior cingulate cortex and precuneus early in the disease, followed by the temporoparietal cortices. On the other hand, atypical presentations of AD such as the posterior variant, logopenic variant, frontal variant, and corticobasal syndrome exhibit hypoperfusion in areas related to symptoms. Additionally, hypoperfusion especially in the precuneus and parietal association cortex can serve as a predictor of progression from mild cognitive impairment to AD. In dementia with Lewy bodies (DLB), the differentiating feature is the presence of hypoperfusion in the occipital lobes in addition to that observed in AD. Hypoperfusion of the occipital lobe is not a remarkable finding, as it is assumed to reflect functional loss due to impairment of the cholinergic and dopaminergic systems rather than degeneration per se. Moreover, the cingulate island sign reflects the degree of AD pathology comorbid in DLB. Frontotemporal dementia is characterized by regional hypoperfusion according to the three clinical types, and the background pathology is diverse. Idiopathic normal pressure hydrocephalus shows apparent hypoperfusion around the Sylvian fissure and corpus callosum and apparent hyperperfusion in high-convexity areas. The cortex or striatum with diffusion restriction on magnetic resonance imaging in prion diseases reflects spongiform degeneration and brain perfusion SPECT reveals hypoperfusion in the same areas. Brain perfusion SPECT findings in dementia should be carefully interpreted considering background pathology.

## Introduction

Brain perfusion single-photon emission computed tomography (SPECT) is a form of nuclear neuroimaging that illustrates alterations in brain perfusion. It can often reveal functional changes in the brain associated with dementia before abnormalities are detected on morphological imaging modalities such as magnetic resonance imaging (MRI). The radiotracers for assessing brain perfusion include N-isopropyl-(^123^I)-p-iodoamphetamine (^123^I-IMP) and technetium-99m hexamethylpropylene amine oxime (^99m^Tc-HMPAO) or ethyl cysteinate dimer (^99m^Tc-ECD). ^123^I-IMP has a higher first-pass extraction than others and a better linearity to perfusion, making it suitable for detecting mild perfusion reduction. Conversely, ^99m^Tc-HMPAO or ^99m^Tc-ECD provides superior image quality because higher doses can be administered [1, 2]. The characteristics of the two main radiotracers, ^123^I-IMP and ^99m^Tc-ECD, are summarized in Table 1.

**Table 1:** Comparison of ^123^I-IMP and ^99m^Tc-ECD

	^123^I-IMP	^99m^Tc-ECD
Pharmacokinetics	Brain accumulation increases gradually after administration, peaking and stabilizing at 20–30 min, as most accumulates in the lungs after intravenous injection and is gradually released into the arterial blood	Brain accumulation is fixed and stabilized in about 2 min as it is metabolized from fat-soluble to water-soluble after passage through BBB and retained in the brain
Emergency scan	Unavailable	Available from ^99^Mo/^99m^Tc generators
*T*_1/2_	13.2 h	6.01 h
Principal energy	159 keV	141 keV
Image quality	Slightly inferior – Limited dosage (111–222 MBq) – Higher energy gamma-rays than 159 keV	Better – Higher dosage (370–740 MBq)
Correlation with perfusion	Better – Higher first-pass extraction fraction (> 90%)	Underestimation in high-flow areas – Lower first-pass extraction fraction (77%)
Quantification	Autoradiography method Graph plot	Patlak plot
Procedure	Scan 20 min after injection	Scan 5 min after injection

^*123*^*I-IMP* N-isopropyl-(123I)-p-iodoamphetamine, ^*99m*^*Tc-ECD* technetium-99 m ethyl cysteinate dimer

^18^F-fluorodeoxyglucose (FDG) positron emission tomography (PET) has good spatial resolution, and some prefer ^18^F-FDG-PET as functional imaging for dementia. However, brain perfusion SPECT is widely employed in dementia practice in Asian countries, such as Japan and Taiwan, as well as in Europe due to its affordability and high diagnostic performance when combined with statistical image analysis [3–5]. Globally, the number of SPECT scanners surpasses that of PET scanners, making SPECT a practical and cost-effective biomarker, particularly in low- and middle-income countries [6, 7].

A major shift is about to occur in the practice of dementia from the conventional approach of diagnosing based on clinical phenotypes and treating with symptom-modifying drugs to the approach of diagnosing based on biomarkers and treating with disease-modifying drugs. Accordingly, brain perfusion SPECT will be required to serve as a biomarker of neurodegeneration. In addition, mismatches between other biomarkers, such as tau, and biomarkers of neurodegeneration are important in assessing copatholgy [8–10].

**Fig. 1:**
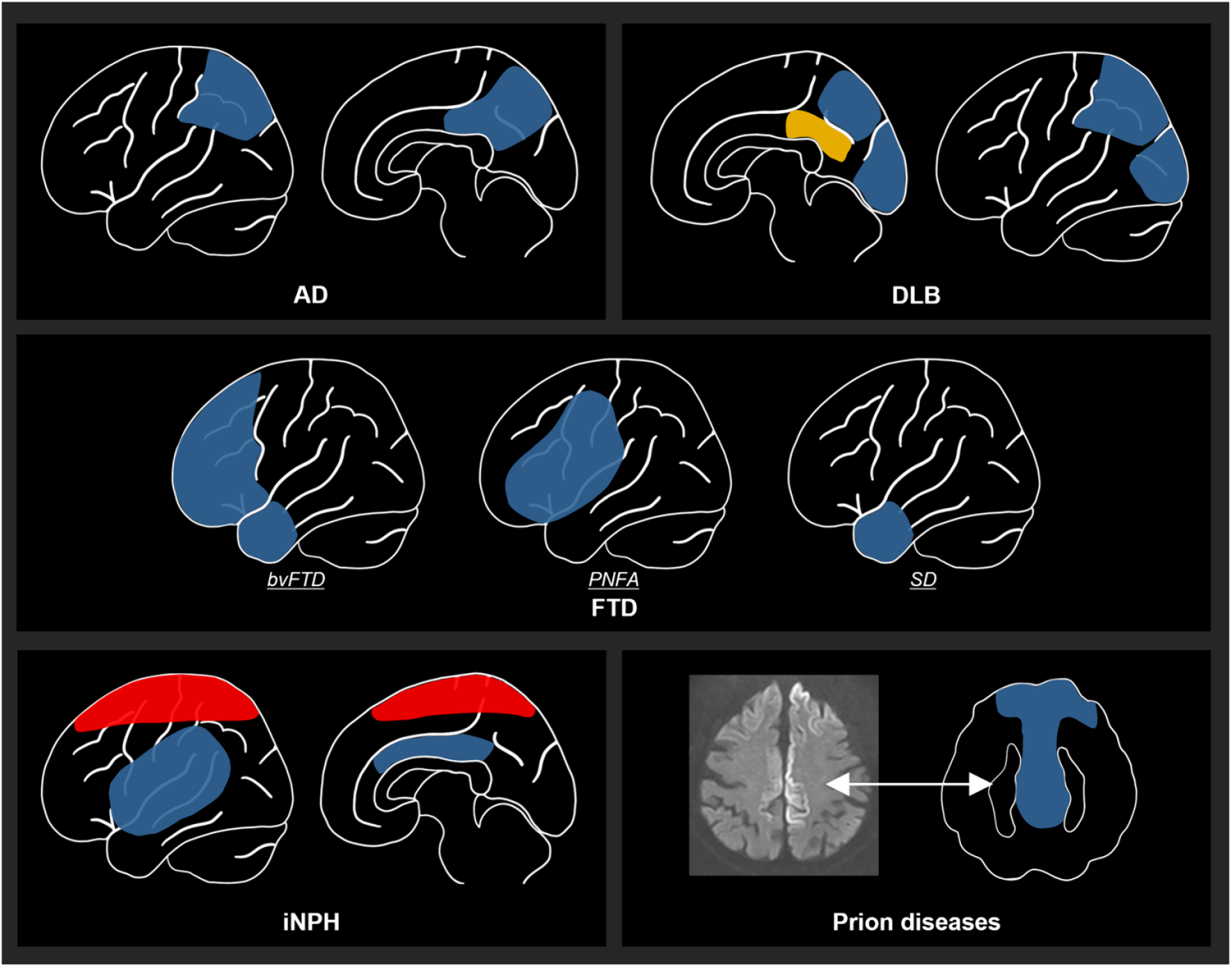
Schematic drawings of typical perfusion patterns. Hypoperfusion areas are illustrated in blue. The yellow area in DLB represents the CIS. The red area in iNPH denotes the CAPPAH sign. Hypoperfusion in prion disease differs from other diseases in that it corresponds to the DWI high-intensity regions rather than specific regions

This review summarizes current knowledge on brain perfusion SPECT in the evaluation of dementia. Figure 1 illustrates the characteristic perfusion patterns of diseases associated with dementia.

## Alzheimer’s disease

First described in 1906, Alzheimer’s disease (AD) is the most common neurodegenerative cause of dementia and is characterized by a slow decline in episodic memory followed by language, visuospatial, and executive difficulties with disease progression [11]. Pathologically, AD is defined by the presence of extracellular senile plaques mainly comprising amyloid beta (Aβ) and intracellular neurofibrillary tangles (NFT) that include tau filaments, resulting in reduced neuronal density due to neuronal death [12]. Neurofibrillary changes typically commence in the transentorhinal cortex and proceed through the medial temporal lobes toward the neocortical association areas in the frontal, parietal, and occipital lobes [13]. Disease-modifying drugs for AD have recently gained attention, increasing the importance of accurate diagnosis [14].

### Brain perfusion SPECT findings

**Fig. 2:**
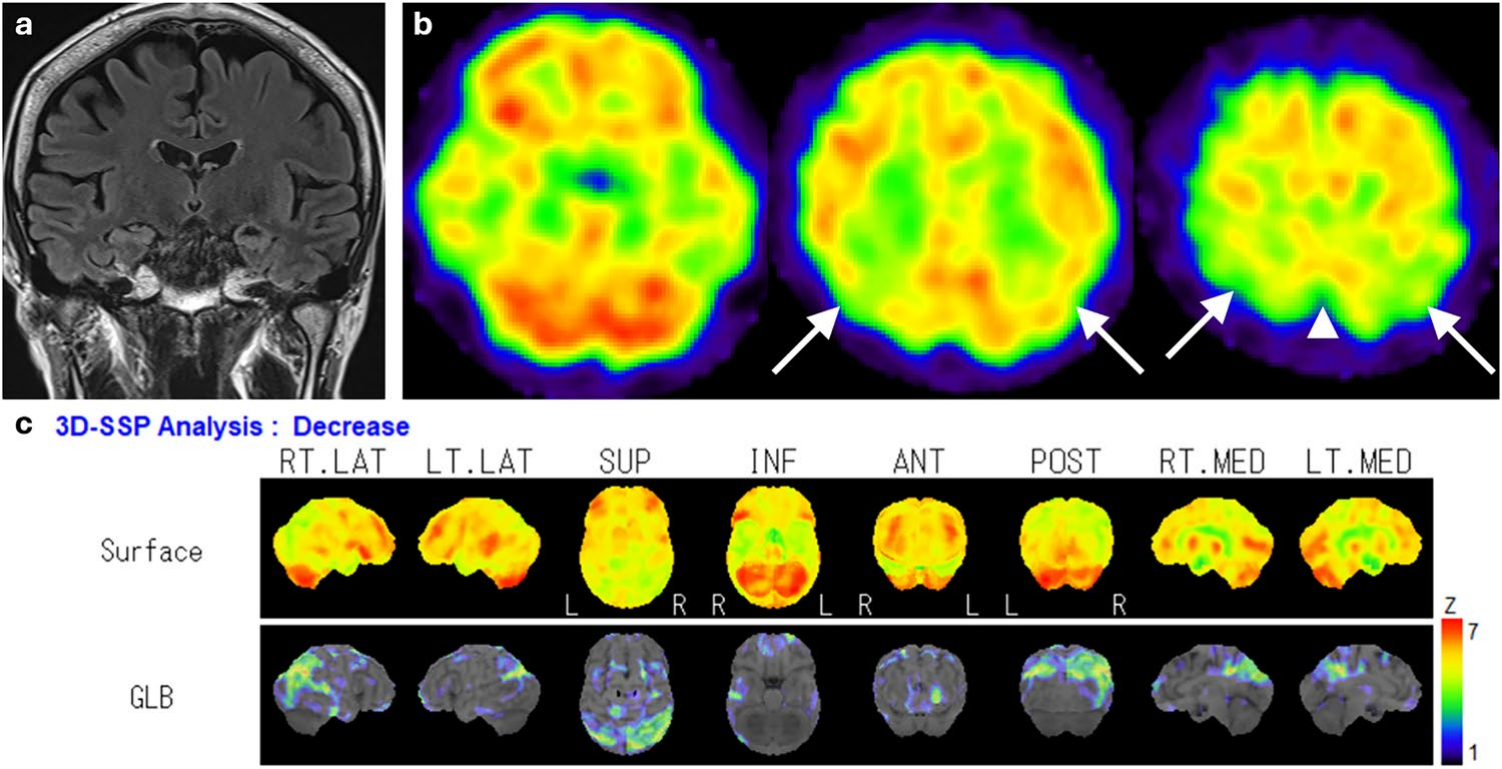
A representative case of AD, a 62-year-old female. **a** Coronal fluid-attenuated inversion recovery (FLAIR) images showed only mild medial temporal lobe atrophy. **b** However, IMP brain perfusion images revealed hypoperfusion in the posterior cingulate gyrus to precuneus (arrowhead) and temporoparietal cortex (arrows) compared with the perirolandic cortex and cerebellum. **c** 3D-SSP analysis demonstrated a similar pattern of hypoperfusion

Hypoperfusion in AD patients is seen in the posterior cingulate cortex and precuneus in the early stages of the disease, followed by bilateral and often asymmetric reductions in temporoparietal cortices (Fig. 2), while frontal cortices are affected in advanced stages. Perfusion is typically preserved in the primary visual and sensorimotor cortices, basal ganglia, thalamus, brainstem, and cerebellum [7, 15, 16].

**Fig. 3:**
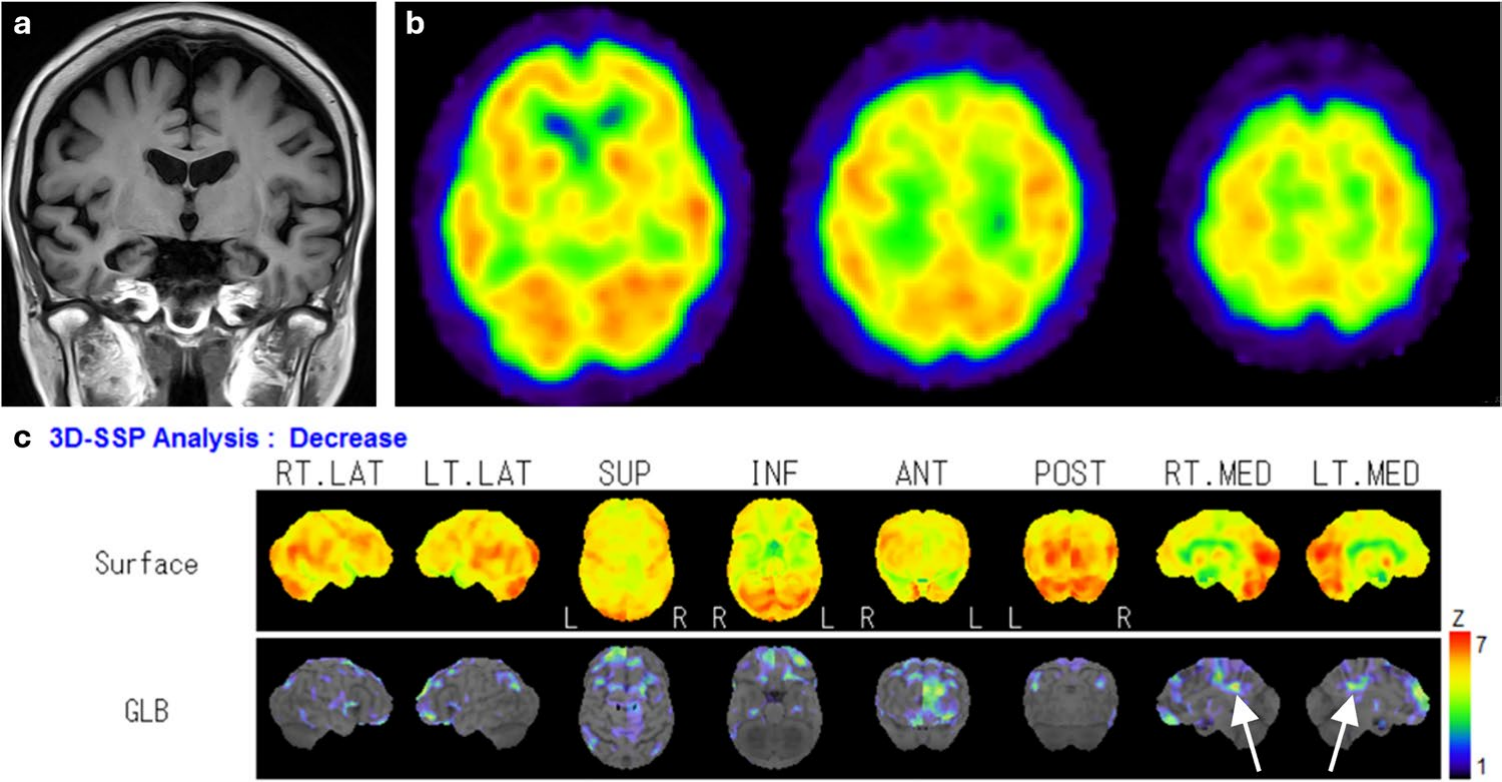
A 76-year-old female with MCI. Elevated CSF pTau and decreased CSF Aβ-42 levels, along with a positive ^18^F-Florbetapir PET scan (not shown), were indicative of AD. FLAIR coronal image revealed bilateral medial temporal lobe atrophy. IMP brain perfusion images exhibited no evident hypoperfusion. However, 3D-SSP analysis detected hypoperfusion in the posterior cingulate gyrus (arrows), consistent with AD

Many studies have evaluated the utility of statistical image analysis and have shown its superior diagnostic performance compared with visual assessment. As the posterior cingulate gyrus and precuneus, which show reduced blood flow early in AD, have as high metabolic activity as the primary visual cortex in healthy individuals at rest, visual assessment of slight hypoperfusion in these areas presents a challenge (Fig. 3) [17]. Three-dimensional stereotactic surface projection (3D-SSP) is a widely used statistical tool and can detect a slight regional perfusion decrease in the posterior cingulate gyrus and precuneus. The 3D-SSP demonstrated an accuracy of 86.2% for discriminating patients with very early-stage AD corresponding to mild cognitive impairment (MCI) from control subjects when analyzing the posterior cingulate gyrus and precuneus. In contrast, visual interpretation exhibited a 10% lower maximum accuracy compared with 3D-SSP [17]. The easy Z-score imaging system (eZIS), which is a subsequently developed statistical tool, enables the use of a common normal database across different institutions through compensation for interinstitutional differences in SPECT camera, collimator, and reconstruction conditions. Z-score is defined as the difference between the sample value of interest and the mean of the distribution, divided by the standard deviation of the distribution. eZIS incorporates the three indices that characterize hypoperfusion in early AD: severity, extent, and ratio. Notably, even when analyzing SPECT images acquired from multiple centers, analysis of the three indices in eZIS showed an accuracy of up to 86% in the differentiation of early AD from normal controls. Additionally, the area under the receiver operating characteristic (ROC) curve reached up to 0.934 [18]. Despite the excellent capabilities of statistical analyses, attention must be paid to the possibility of pseudohypoperfusion, especially in cingulate gyrus, arising from inaccurate stereotactic transformation due to atrophy or enlargement of ventricles and sulci [19].

Differences have been observed between the brain perfusion SPECT findings of early- and late-onset AD. In late-onset AD, findings typical of AD could be less prominent and should be interpreted with caution. With the use of 3D-SSP, Hanyu et al. compared the brain perfusion of 31 cases aged less than 70 years and 48 cases aged 70 years or older who met the criteria for probable AD according to the National Institute of Neurological and Communicative Disorders and Stroke and the Alzheimer’s Disease and Related Disorders Association (NINCDS–ADRDA) criteria [20]. Although both groups showed reduced accumulation in the posterior cingulate cortex, precuneus, and parietal lobes, the younger group exhibited a greater reduction in these areas, and the older group presented additional hypoperfusion in the medial frontal and medial temporal lobes [20]. These results might be influenced by the increase in mixed pathology with age [21]; in addition, non-AD pathologies that clinically mimic AD, such as argyrophilic grain dementia and senile dementia of the NFT type, are more common in the elderly [22–24]. However, similar results have been reported by a study involving ^11^C-Pittsburgh compound B-PET-positive patients with probable AD according to the National Institute on Aging–Alzheimer’s Association (NIA–AA) diagnostic criteria [25]. Moreover, AD patients with hippocampal sparing neurofibrillary pathology were found to be younger than those with typical or limbic-predominant neurofibrillary pathology, and exhibited higher NFT densities in cortical areas [26]; MRI studies have revealed a predominant parietotemporal lobe atrophy in early-onset cases [27, 28]. Differences in SPECT findings by the age of onset can be attributed in part to the different distribution and density of AD pathology.

**Fig. 4:**
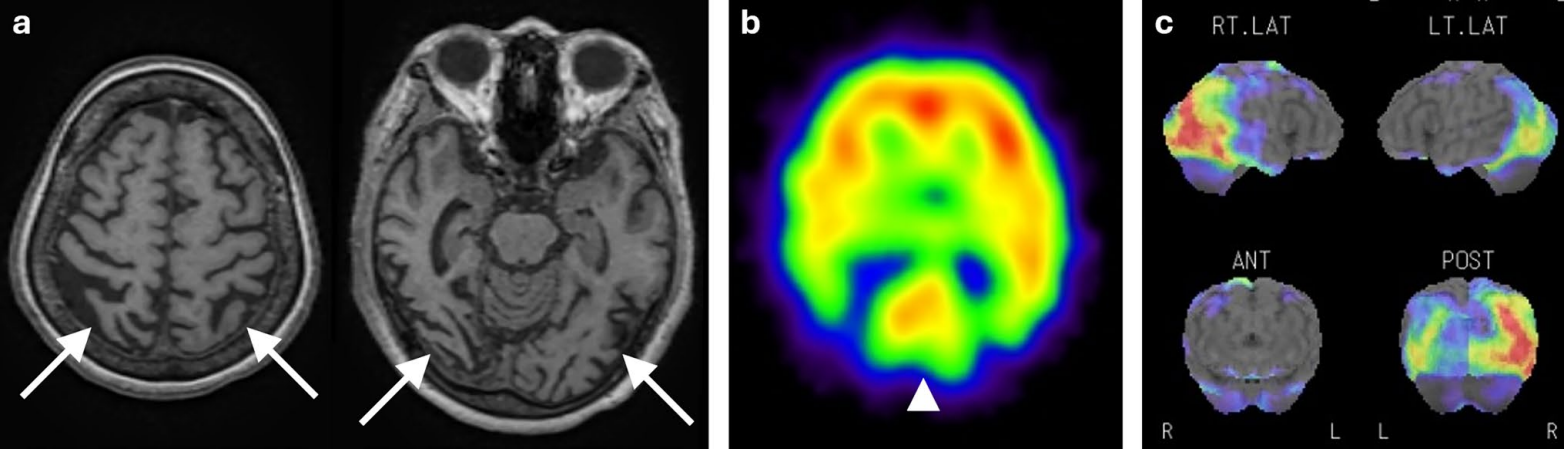
A 73-year-old female with posterior cortical atrophy with left hemispatial neglect, visual agnosia, alexia with agraphia, prosopagnosia, and simultanagnosia. Elevated CSF pTau and decreased CSF Aβ-42 levels indicated AD as background pathology. **a** T1-weighted image (T1WI) showed right-dominant atrophy in the parietal, occipital, and temporal lobes (arrows). **b** IMP brain perfusion images demonstrated a CIS-like finding (arrowhead). **c** 3D-SSP analysis revealed hypoperfusion consistent with atrophic regions. This case was presented in a previous study [31]

Atypical perfusion patterns can be observed in several atypical presentations of AD, including posterior variant, logopenic variant, frontal variant, and corticobasal syndrome. These variants are characterized by a relative preservation of memory with characteristic symptoms accompanied by regional atrophy or hypoperfusion in related areas [29–31]. The posterior variant of AD presents as a posterior cortical atrophy and is characterized by progressive decline in visuospatial, visuoperceptual, literacy, and praxis skills. This variant shows atrophy of the occipital and parietal lobes followed by areas in the temporal lobe, with less hippocampal atrophy than typical AD. Hypoperfusion on SPECT presents in the same areas as these morphological changes (Fig. 4). Notably, although AD is the most common underlying pathology of posterior cortical atrophy, some cases are attributable to other causes, such as corticobasal degeneration, dementia with Lewy bodies (DLB), prion disease, and subcortical gliosis [32].

**Fig. 5:**
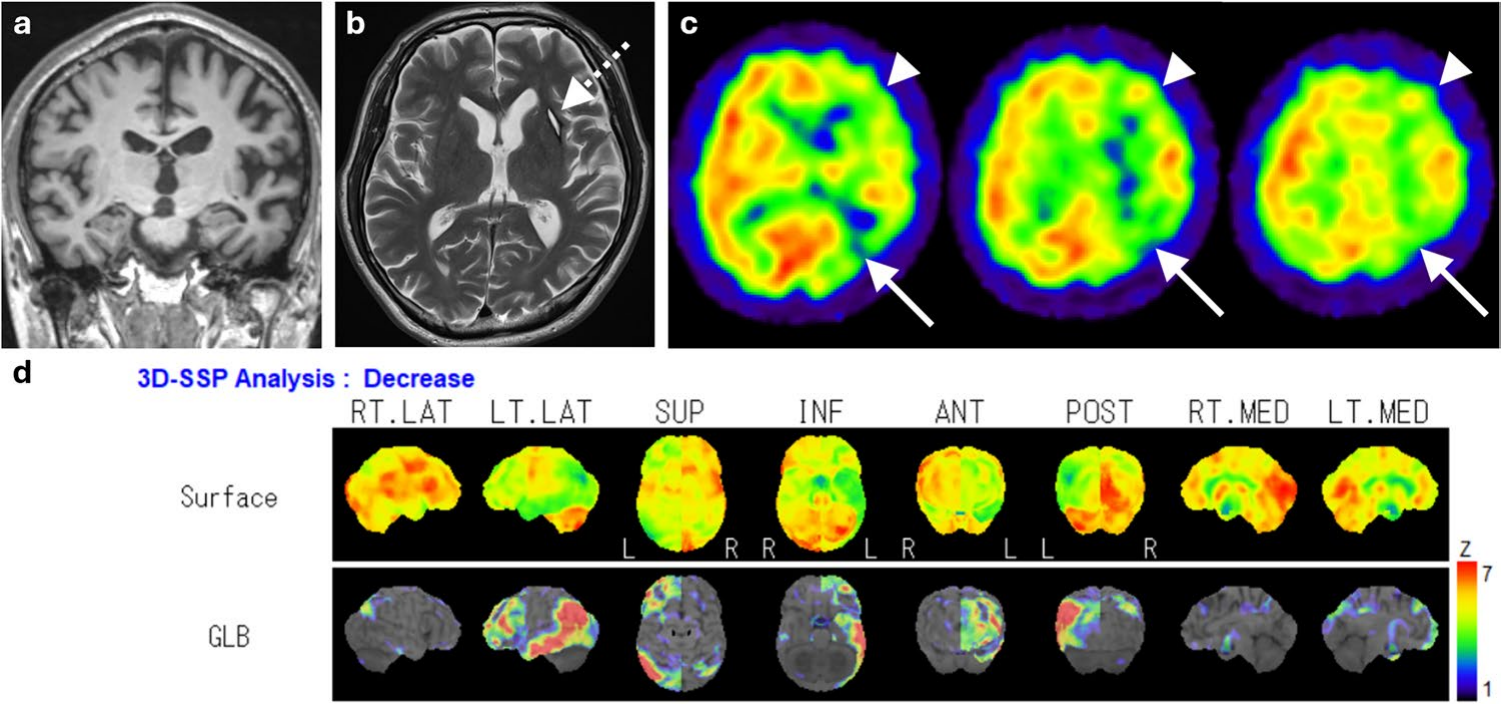
A 60-year-old male with logopenic progressive aphasia. Elevated CSF pTau and decreased CSF Aβ-42 levels indicated AD as a background pathology. **a** Coronal T1WI revealed the enlargement of the left Sylvian fissure. **b** Axial T2WI demonstrated a post-hemorrhagic change in the left putamen (dashed arrow). **c** IMP brain perfusion images. **d** 3D-SSP analysis revealed the left-sided posterior peri-Sylvian and temporoparietal hypoperfusion (arrows), consistent with logopenic progressive aphasia. Hypoperfusion in the left frontal lobe (arrowheads) was attributed to a remote effect of the left putaminal hemorrhage

The logopenic variant of AD presents as the logopenic progressive aphasia, which shows word-finding difficulties and impaired repetition of sentences and phrases. This variant demonstrates asymmetric, classically left-sided, posterior peri-Sylvian, and temporoparietal atrophy. Hypoperfusion is seen in the same regions as atrophy, specifically involving the left inferior parietal lobule and left posterior superior and middle temporal gyri, including the expected Wernicke area (Fig. 5) [33].

The frontal variant of AD is a rare variant and characterized by early and predominant behavioral deficits and personality changes. However, Alzheimer’s pathology is detected in only a small proportion of patients with prominent frontal behavioral symptoms. Two phenotypes on neuroimaging, including MRI, PET, and SPECT, were observed across reported frontal variant cases. These phenotypes include an AD-like pattern with relative frontal sparing and a relatively more behavioral frontotemporal dementia (FTD)-like pattern with both posterior and anterior involvement, with the former being the most prevalent [30].

AD accounts for 23% of the underlying pathology of corticobasal syndrome. In addition to atrophy around the central sulcus, there is atrophy in regions corresponding to the underlying pathology in corticobasal syndrome. For example, corticobasal syndrome due to AD shows atrophy extend into temporoparietal cortex and precuneus [34]. Similar findings have been reported in molecular imaging. Corticobasal syndrome is characterized by hypoperfusion/hypometabolism in the frontoparietal regions on the contralateral side to the dominant symptomatic side, which comes more prominent toward the posterior regions including the parietal lobe and posterior cingulate gyrus in cases where AD is the underlying pathology [34, 35].

### Clinical utility

The NIA–AA has introduced the ATN classification system for grouping of various AD biomarkers based on the pathologic process they measure. Biomarkers of Aβ plaques (labeled as “A”) include cortical amyloid PET ligand binding or low cerebrospinal fluid (CSF) Aβ-42. Those of fibrillar tau (labeled as “T”) consist of elevated CSF phosphorylated tau (pTau) and cortical tau PET ligand binding. As for neurodegeneration or neuronal injury (labeled as “N”), its biomarkers comprise CSF total tau, ^18^F-FDG-PET hypometabolism, and atrophy on MRI [36].

Brain perfusion SPECT, although not included in “N” biomarkers, substitutes for ^18^F-FDG-PET as an indicator of neurodegeneration in clinical practice due to its better accessibility [3]. Brain perfusion SPECT studies have revealed findings associated with neurodegeneration in AD, in the same cortical areas as those observed in ^18^F-FDG-PET [3, 15, 37]. In a meta-analysis, the sensitivity and specificity for distinguishing AD from normal controls using brain perfusion SPECT were reported as 80% and 85%, respectively; these values were comparable to those observed for ^18^F-FDG-PET (90% and 89%) [38]. For pathologically confirmed AD, brain perfusion SPECT exhibited a sensitivity of 94%, specificity of 85%, and an overall accuracy of 90% [39]. Moreover, the clinical diagnosis of “probable” AD based on NINCDS–ADRDA criteria was associated with an 84% likelihood of pathological diagnosis of AD; a positive SPECT scan increased the likelihood of AD to 92%, and a negative SPECT scan reduced the likelihood to 70% in a logistic regression analysis [40]. In the same study, SPECT also proved valuable when the clinical diagnosis was “possible” AD, with a likelihood of 67% without SPECT, 84% with a positive SPECT, and 52% with a negative SPECT [40].

Brain perfusion SPECT holds potential for the prediction of progression from MCI to AD. Studies have indicated that individuals with MCI who progress to AD exhibit hypoperfusion primarily in the posterior cingulate gyrus, precuneus, and parietal association cortex [37, 41–48]. Notably, hypoperfusion in the posterior cingulate gyrus has also been observed in MCI patients not progressing to AD within 3 years [46]. Therefore, some investigations suggested the possibility of hypoperfusion in the precuneus and parietal association cortex being a better predictor of MCI progression to AD compared with that in the posterior cingulate gyrus [45–47]. This finding aligns with the hypothesis that hypoperfusion in the posterior cingulate gyrus represents a remote effect of medial temporal lobe degeneration, which is an early-AD pathology, while hypoperfusion in the parietotemporal lobe corresponds to a more advanced pathology [16, 46]. In a meta-analysis, SPECT demonstrated a sensitivity of 84% and specificity of 70% in the prediction of progression from MCI to AD. The corresponding values for ^18^F-FDG-PET were 89% and 85%. Various analysis methods were employed in the studies encompassed by the meta-analysis and heterogeneity among the studies was suggested, which implies the need for careful comparison [49]. Another meta-analysis reported the pooled sensitivity and specificity for conversion of MCI to AD, with values of 76 and 74%, respectively, for ^18^F-FDG-PET and 78 and 64% for SPECT [50].

## Dementia with Lewy bodies

DLB is the second most common cause of neurodegenerative dementia after AD [51]. In the revised criteria for the clinical diagnosis of probable and possible DLB, core clinical features include fluctuating cognition with pronounced variations in attention and alertness, recurrent visual hallucinations, rapid eye movement sleep behavior disorder, and parkinsonism [52]. Pathologically, DLB presents the deposition of α-synuclein in Lewy bodies (LB) and neurites throughout the brain often with variable degree of coexisting Alzheimer pathology [53–55].

The revised criteria for the clinical diagnosis of probable and possible DLB incorporate both core clinical features mentioned above and indicative biomarkers as essential components for diagnosis. Supportive biomarkers are positioned as an aid in the diagnosis of DLB, with brain perfusion SPECT being included in this category. Focusing on imaging tests, indicative biomarkers consist of dopamine transporter imaging and ^123^iodine-meta-iodobenzylguanidine myocardial scintigraphy. Supportive biomarkers comprise MRI/CT and brain perfusion SPECT/^18^F-FDG-PET with emphasis on the differentiation from AD. Specifically, MRI/CT scans highlight the relative preservation of the medial temporal lobe. In addition, generalized low uptake with reduced occipital activity on brain perfusion SPECT or ^18^F-FDG-PET is considered as an supportive biomarker, and the cingulate island sign (CIS) on ^18^F-FDG-PET that reflects sparing of the posterior cingulate cortex relative to the precuneus plus cuneus is also included [52].

### Brain perfusion SPECT findings and clinical utility

**Fig. 6:**
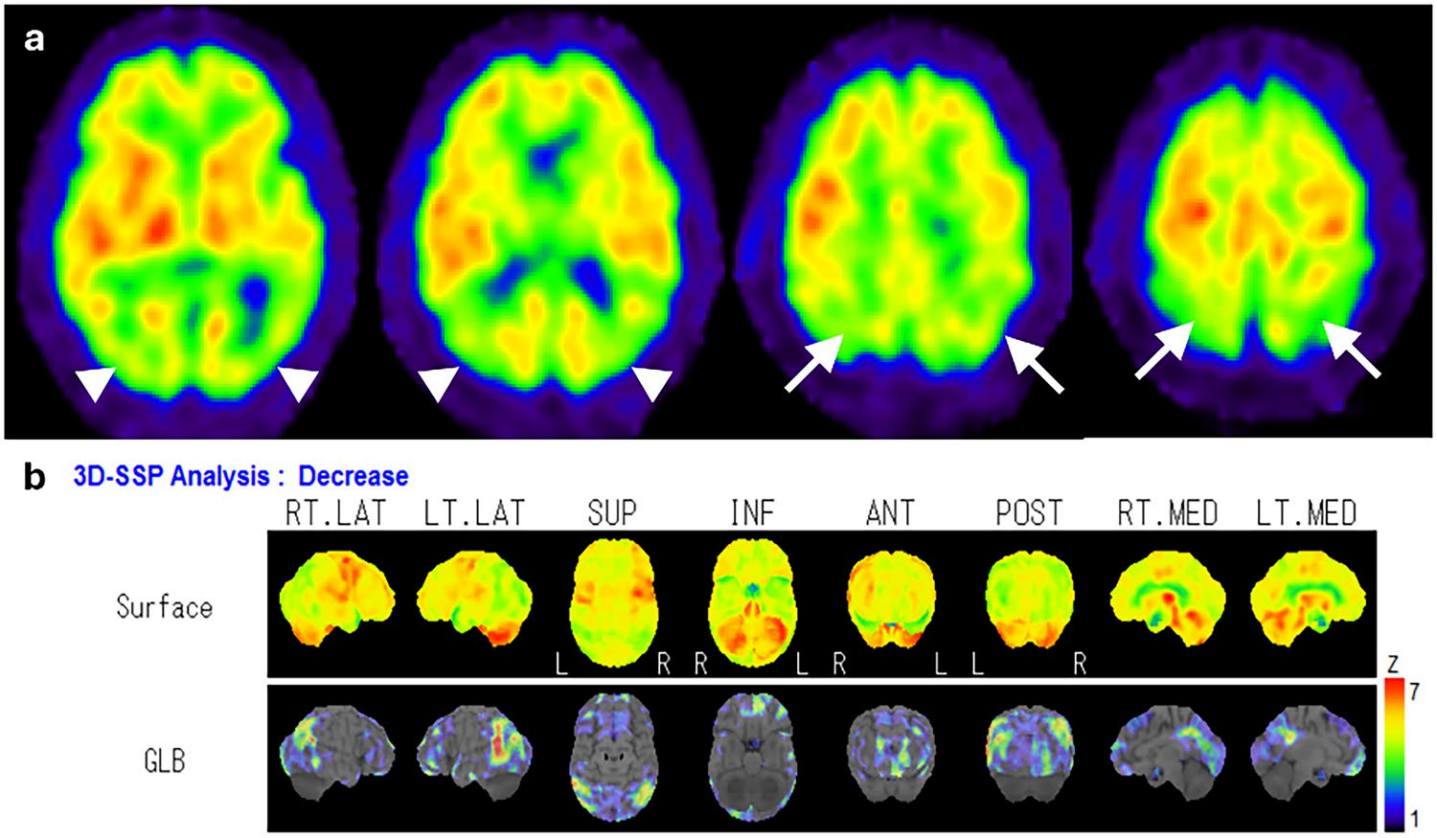
A representative case of DLB, a 69-year-old male presented with cognitive impairment, visual hallucinations, and parkinsonism. There was decreased striatal accumulation on ^123^I-iofulupane SPECT and decreased myocardial accumulation on ^123^I-MIBG scintigraphy (not shown). **a** IMP brain perfusion images and **b** 3D-SSP analysis revealed hypoperfusion in the occipital lobe (arrowheads) in addition to the posterior cingulate gyrus to precuneus and temporoparietal cortex (arrows), consistent with DLB. The decreased accumulation in the posterior cingulate gyrus was evident, suggesting the possibility of concomitant AD pathology

In addition to hypoperfusion in the parietotemporal lobe and posterior cingulate gyrus similar to AD, hypoperfusion in the occipital lobe is a distinguishing feature in DLB (Fig. 6) [56–60]. Although hypoperfusion of the occipital lobe serves as a supportive biomarker for DLB, it is not a prominent finding. In DLB, the reduction of accumulation in the occipital lobe is less than that in the parietal lobe [61]. Given that the occipital lobe typically exhibits higher accumulation in normal cases, even with reduction due to DLB, there is comparable or slightly higher accumulation in the occipital lobe compared to other sites [58, 61], and it should be noted during visual evaluation. Pathologically LB rarely involve the occipital lobe and the decreased accumulation in the occipital lobe is speculated to be a dysfunction resulting from impairment of the cholinergic and dopaminergic systems [57, 61] and an ^18^F-FDG-PET study in autopsy cases revealed the correlation of hypometabolism in the occipital lobe with substantia nigra neuronal loss [62]. The mild hypoperfusion in the occipital lobe may support this theory. Statistical image analysis proves to be highly useful in the evaluation of occipital perfusion, with reported sensitivities ranging from 65 to 85% and specificities from 71 to 87% when distinguishing DLB from AD [57–60]. Within the occipital lobe, the evaluation of the medial occipital lobe is particularly important to differentiate DLB from AD. This point was supported by an ROC analysis, with an area under the curve of 0.905 in the medial occipital lobe compared with the values of 0.749 and 0.609 in the lateral and inferior occipital lobes, respectively [59]. Although no study has directly compared radionuclides for the detection of occipital hypoperfusion in DLB, studies utilizing ^123^I-IMP have demonstrated higher diagnostic performance compared with those using ^99m^Tc-HMPAO or ^99m^Tc-ECD [57–60]. This finding may be attributed to the high correlation of perfusion and accumulation in high-flow areas when using ^123^I-IMP. Additionally, the association between reduced occipital lobe accumulation and visual hallucinations is controversial [58, 63, 64].

Hypoperfusion of the occipital lobe is sometimes observed in patients who are clinically considered to have AD [57]. There is a study on ^18^F-FDG-PET involving 52 autopsy cases who were clinically considered to have AD or amnestic MCI. The pathology of these cases consisted of pure-AD (21 cases), AD with LB neuropathologic change (AD–LB; 24 cases), and pure-LB (7 cases). The pure-LB group exhibited a significantly reduced accumulation in the occipital lobe and AD–LB patients with high substantia nigra neuronal loss displayed hypometabolism in the occipital lobe. The reduced accumulation in the occipital lobe in clinical AD may suggest pathological pure-LB or AD–LB. Notably, only a few cases of AD–LB showed the DLB-like hypometabolic pattern [62].

The CIS is a supportive biomarker in ^18^F-FDG-PET and is also useful in SPECT. Relative metabolic retention in the posterior cingulate gyrus on ^18^F-FDG-PET was reported as early as 1997 [65]. Lim et al. reported a sensitivity ranging between 62 and 86% and specificity of 100% when visually assessing the CIS to differentiate DLB from AD, with a diagnostic accuracy of 78% for the ratio of accumulation in the posterior cingulate gyrus to that in precuneus and cuneus [66]. Although a study reported the difficulty to detect CIS by brain perfusion SPECT, it can be inferred that the statistical image analysis using appropriate regions of interest can achieve a diagnostic performance as good as that of ^18^F-FDG-PET. This inference arises from studies comparing AD, DLB, and normal groups. Although the reduction of accumulation in brain perfusion SPECT was relatively smaller compared to that in ^18^F-FDG-PET, the areas of reduced accumulation were the same for both modalities [61, 67, 68]. Indeed, Imabayashi et al. achieved a diagnostic accuracy of 80% in discriminating DLB from AD; they utilized the ratio of decreased accumulation in the posterior cingulate gyrus and precuneus within the early-AD-specific volume of interest, which presented hypoperfusion in the early-AD group compared with healthy controls [69]. The regions of interest were further improved, achieving a positive diagnosis rate of 84.6% by using the total Z-score of the disease-specific region of DLB as the denominator and the total Z-score of the disease-specific region of AD excluding the disease-specific region of DLB as the numerator [70]. The CIS score obtained using these regions of interest is calculated in eZIS and applicable in clinical practice. Moreover, Honda et al. noted that the CIS score can produce false-positive results in the presence of reduced accumulation in the occipital lobe in AD; they reported a 93% accuracy for the differentiation of DLB and AD through the analysis of reduced accumulation in the parahippocampal regions, which is characteristic of AD, in combination with reduced accumulation in the occipital gyri, which is characteristic of DLB [71].

The CIS also provides insights into AD pathology coexisting with DLB. Graff-Radford et al. found that CIS in ^18^F-FDG-PET was observed in DLB patients regardless of amyloid PET results. In a further analysis of 10 autopsy cases, which comprised 8 clinically diagnosed DLB cases and 2 clinically diagnosed AD cases, the CIS was more prominent in patients with lower Braak NFT staging [72]. The temporal changes of the CIS were also examined using brain perfusion SPECT. The sign was indistinct in prodromal DLB but became more evident in mild DLB, reaching its maximum when the Mini-Mental State Examination score was around 22. The sign became unclear again with the disease progression, which was considered to reflect an increase in AD-type NFT pathology with disease progression [2]. In addition, cognitive decline was more advanced over 2 years in the mild DLB group when the CIS was unclear, suggesting a concomitant AD pathology [2]. This result is consistent with previous studies showing more rapid cognitive decline in DLB with AD pathology compared with pure DLB [73]. The finding that the CIS tends to be obscured in prodromal or advanced DLB seems to be helpful in the interpretation of brain perfusion SPECT. Moreover, if a patient with DLB presents with decreased accumulation in the posterior cingulate gyrus and the CIS is unclear, coexistence of AD pathology is possible. The evaluation of brain atrophy is also important. An autopsy study demonstrated that DLB patients exhibited atrophy and ventricular enlargement comparable to normal controls. In contrast, mixed DLB/AD patients displayed severe brain atrophy, including atrophy in the hippocampus, parahippocampus, and parietal temporal lobe, which was similar in distribution to that seen in AD [74].

A potential pitfall is that the CIS can also be observed in posterior cortical atrophy, despite the underlying pathology primarily being AD (Fig. 4) [75]. In posterior cortical atrophy, NFT pathology often relatively spares the medial temporal lobe. Additionally, in DLB, CIS negatively correlates with medial temporal lobe atrophy [76]. Hence, CIS in DLB and posterior cortical atrophy may indicate a preservation of the posterior limbic circuitry [75, 76].

Parkinson’s disease and Parkinson’s disease dementia, along with DLB, are considered on the spectrum of LB disease, and there are similarities in brain perfusion SPECT findings [77]. Parkinson’s disease dementia is diagnosed when dementia appears more than one year after the onset of parkinsonism, but brain perfusion SPECT findings are similar to those of DLB and undistinguishable [78, 79]. In addition, Parkinson’s disease exhibits decreased accumulation in the frontal and occipital lobes, which overlaps with findings observed for DLB [79–81].

## Frontotemporal dementia

FTD is an umbrella term for a spectrum of clinical syndromes characterized by progressive decline in behavior, executive function, or language. This condition is classified into three types: behavioral variant FTD, semantic dementia (SD), and progressive nonfluent aphasia (PNFA) [82]. SD and PNFA correspond to semantic variant primary progressive aphasia (PPA) and nonfluent/agrammatic variant PPA, respectively [83]. Frontotemporal lobar degeneration (FTLD), on the other hand, is an umbrella pathological term that refers to a group of neurodegenerative diseases characterized by neuronal cell loss mainly in the frontal and temporal lobes, and aggregation and accumulation of specific proteins within the remaining neuronal and glial cells. Tau, TAR DNA-binding protein of 43 kDa (TDP-43), and fused in sarcoma (FUS) account for approximately 45%, 50%, and 5% of the accumulated proteins, respectively, and form the three major pathological subtypes: FTLD-tau, FTLD-TDP, and FTLD-FUS [84, 85]. The rarer subtypes include FTLD-ubiquitin proteasome system and FTLD-no inclusions. FTLD-tau includes Pick’s disease, CBD, progressive supranuclear palsy (PSP), argyrophilic grain disease (AGD), and globular glial tauopathy [86]. FTD usually has FTLD as its background. In this review, the term FTD is used when the focus is on the clinical syndrome, whereas FTLD is employed to refer to the pathological condition [86, 87].

### Brain perfusion SPECT findings in bvFTD

**Fig. 7:**
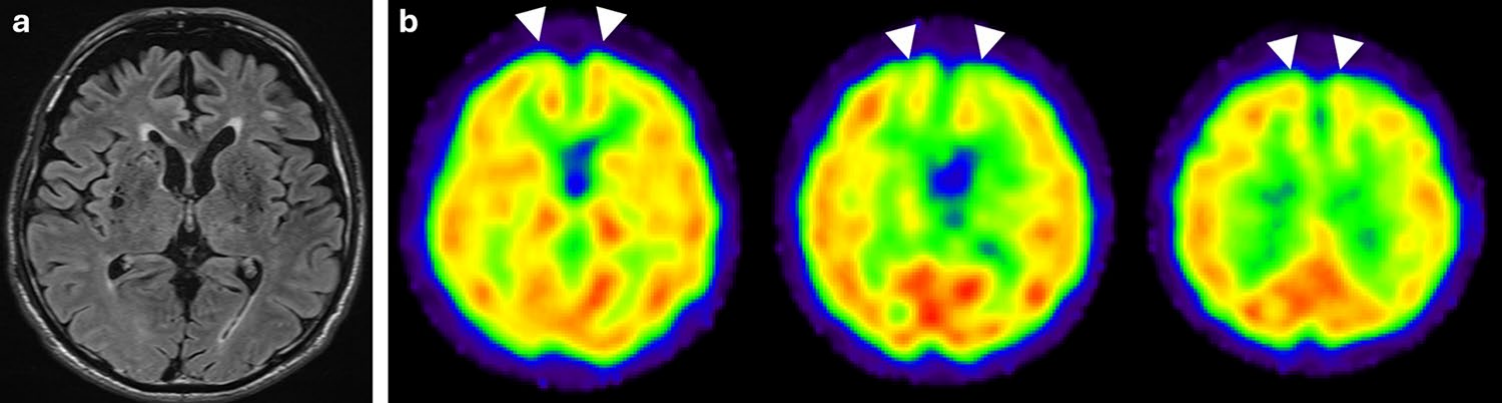
A 79-year-old male with bvFTD. **a** Axial FLAIR images revealed mild atrophy of the frontal lobes. **b** IMP brain perfusion images demonstrated bilateral frontal hypoperfusion consistent with bvFTD (arrowheads)

In each type of FTD, symptoms correspond well the areas of brain degeneration, as reflected by atrophy on MRI and decreased accumulation on brain perfusion SPECT or ^18^F-FDG-PET. bvFTD is characterized by changes in personality and behavioral abnormalities, such as apathy, disinhibition and obsessive–compulsive behaviors, and loss of insight [88]. As the disease advances, bvFTD can also manifest with language impairment and can be associated with parkinsonism and motor neuron disease [87]. bvFTD is characterized by atrophy and reduced accumulation in the frontal and anterior temporal lobes (Fig. 7), which are included in the requirements for probable FTD in the international consensus criteria for bvFTD [89, 90]. However, variations exist in the patterns of reduced accumulation in individual cases. The patterns can range from predominantly in the frontal lobes to predominantly in the temporal lobes, and sometimes involve the parietal lobes [91, 92]. MRI has revealed similar variations in the pattern of atrophy [93–95]. Clinical characteristics differ according to these phenotypes, with the frontal phenotype typically exhibiting poorer executive and language performance, and the temporal phenotype tending to demonstrate more pronounced episodic memory impairment [91]. There is a study of brain atrophy on MRI of autopsy cases of bvFTD. The pathology of bvFTD consisted of CBD, PSP, AGD, and various other FTLDs, as well as AD accounting for 13% of the cases. Mixed pathology was also commonly observed. The pattern of brain atrophy was varied depending on the background pathology [94]. The variations in hypoperfusion/hypometabolism seen in bvFTD may reflect this diverse background pathology. Furthermore, atrophy of the bilateral fronto-insular, anterior to mid-cingulate cortex, amygdala, and striatum was common regardless of pathology and could be degenerative regions closely related to behavioral abnormalities [94].

### Brain perfusion SPECT findings in SD

**Fig. 8:**
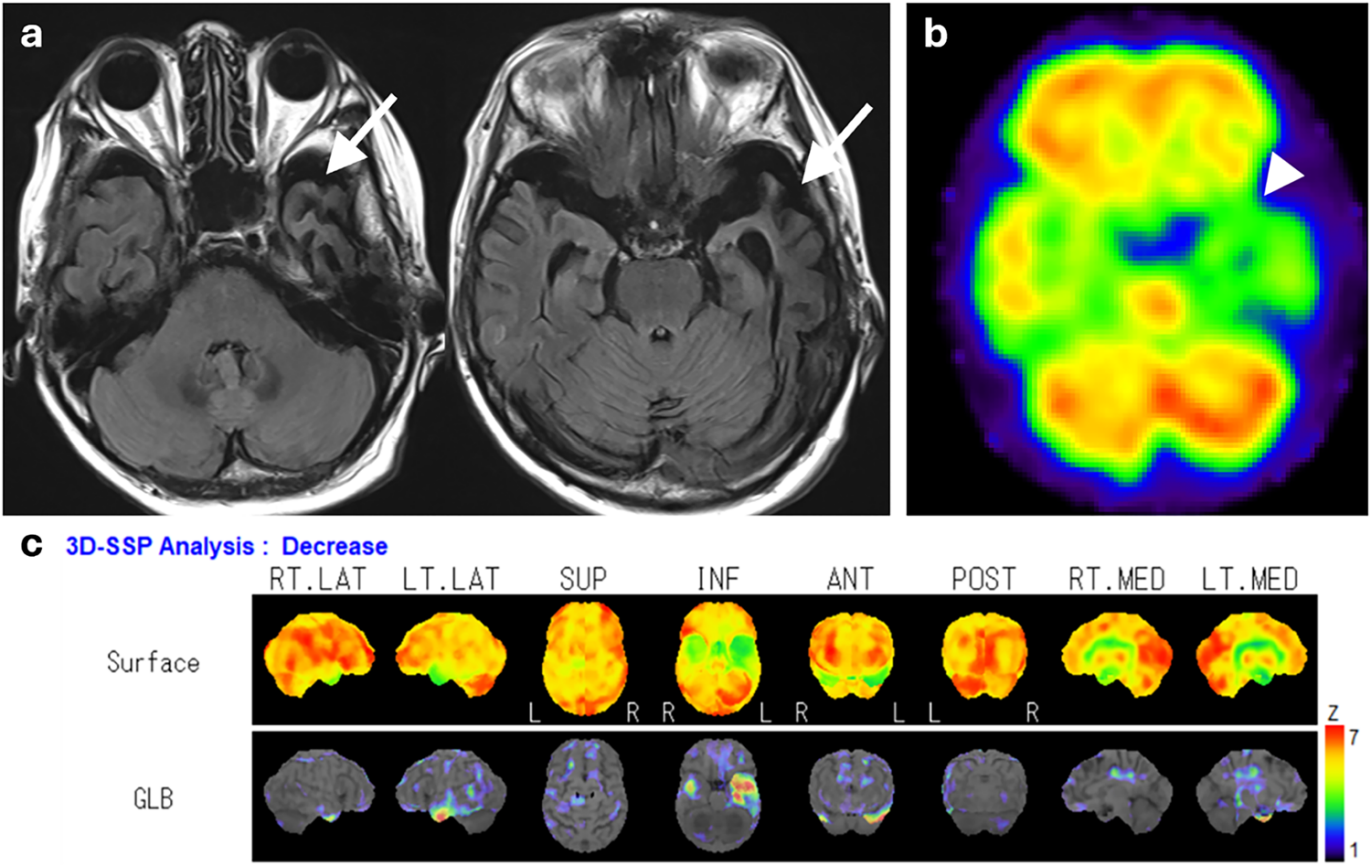
A 60-year-old female with SD. **a** Axial FLAIR images displayed atrophy in the anterior left temporal lobe (arrows), and **b** IMP brain perfusion images (arrowhead) and **c** 3D-SSP analysis revealed hypoperfusion in the same area, consistent with SD

SD is characterized by a progressive cognitive and language deficit, primarily involving comprehension of words and related semantic processing. Patients with SD lose the meaning of words, usually nouns, but retain fluency, phonology, and syntax [96]. The main pathology of SD is FTLD-TDP, especially TDP type C, but FTLD-tau and AD have also been reported [97]. SD is characterized by atrophy of the anterior temporal lobe and hypoperfusion/hypometabolism (Fig. 8) [33, 87, 98]. These neuroimaging features are part of the diagnostic criteria of Gorno-Tempini et al. [83]. A meta-analysis of voxel-based morphometry of SD showed a left-sided predominant atrophy. Gray matter volume reduction was seen in the bilateral fusiform and inferior temporal gyri, extending to the medial portion of the temporal lobes (including the amygdala and parahippocampal gyri), as well as in the left temporal pole, left middle temporal gyrus, and left caudate [99]. In contrast, patients with SD accompanied with prosopagnosia showed right-dominant atrophy [100]. However, over time, atrophy extended to the temporal lobe contralateral to the dominant side of the atrophy, and atrophy progressed in the frontal lobe [101, 102]. In addition, as a differentiation point from AD, which also causes atrophy of the temporal lobe, atrophy in SD affects the entire temporal lobe, including the lateral temporal lobe, and is particularly prominent in the anterior region [103].

### Brain perfusion SPECT findings in PNFA

**Fig. 9:**
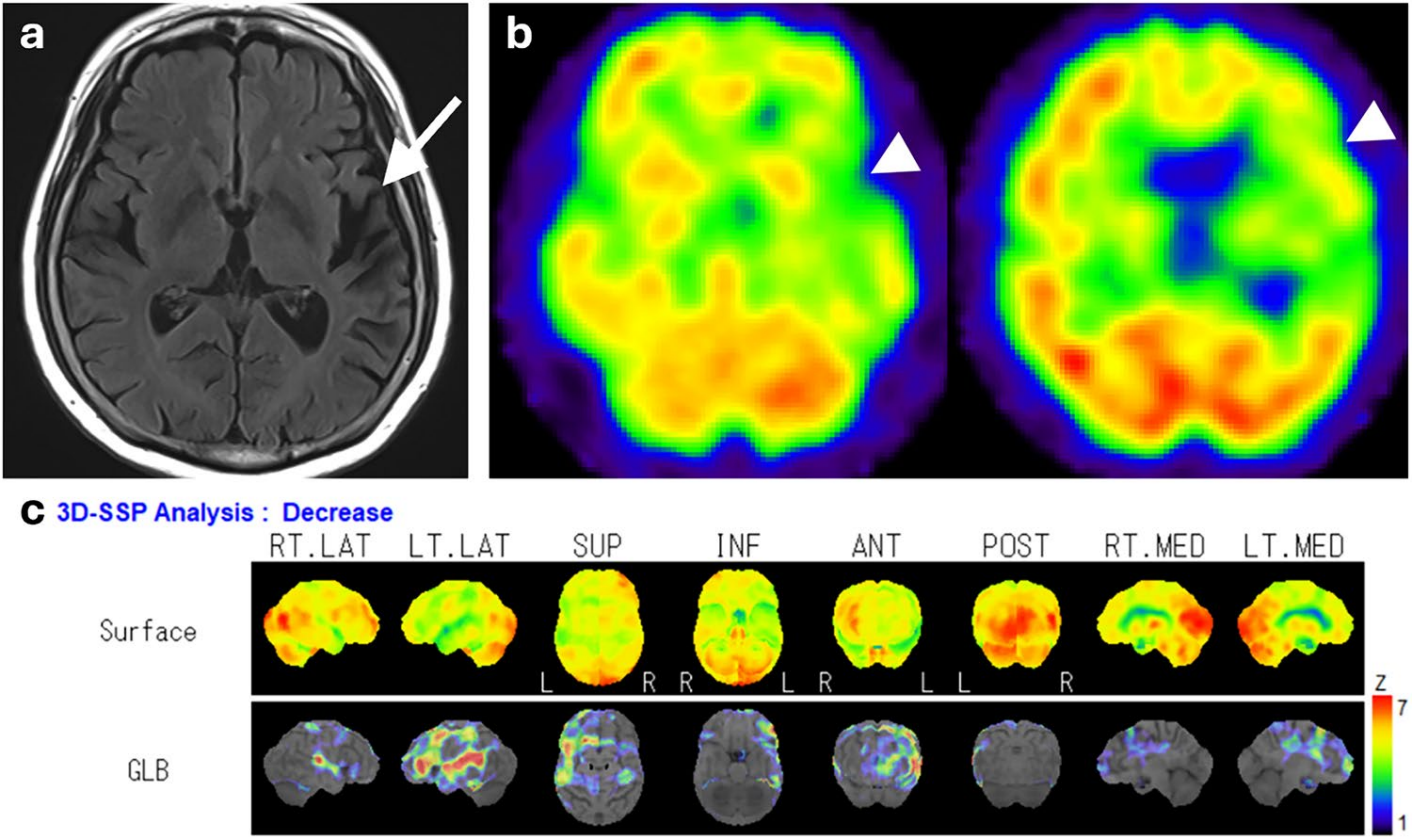
A 66-year-old female with PNFA. **a** Axial FLAIR images showed a subtle enlargement of the left Sylvian fissure (arrow). **b** IMP brain perfusion images and **c** 3D-SSP analysis demonstrated hypoperfusion in the posterior left frontal lobe extending to regions around the left Sylvian fissure, consistent with PNFA (arrowheads)

In the diagnostic criteria of Gorno-Tempini et al., agrammatism in language production and effortful, halting speech with inconsistent speech sound errors, and distortions (apraxia of speech) are core features of PNFA, and the requirement is that one of them should be fulfilled [83]. Agrammatism typically comprises short, simple phrases and omissions of grammatical morphemes. In addition, two of the following must be met for diagnosis of PNFA: impaired comprehension of syntactically complex sentences, spared single-word comprehension, and spared object knowledge [83]. Background pathology in PNFA is predominantly FTLD-tau, but FTLD-TDP and AD have also been found in some cases [104]. Four-repeat tauopathy is common among FTLD-tau, with CBD and PSP accounting for a large proportion [105]. Moreover, a number of PNFA cases present with motor problems consistent with the diagnosis of corticobasal syndrome or PSP in the disease course [106]. The diagnostic criteria of Gorno-Tempini et al. emphasize predominant left posterior fronto-insular atrophy and hypoperfusion/hypometabolism (Fig. 9) [83]. More specifically, atrophy and decreased accumulation in PNFA have been observed in the left posterior frontal lobe including the inferior (including Broca’s area), middle and superior premotor gyri, as well as in the left insula, left superior temporal lobe, and other regions in the left frontal and parietal lobes, accompanied with dilatation of the Sylvian fissure [106–113]. Among these sites, the areas showing atrophy and decreased accumulation corresponding to the two core features are distinct from each other. Apraxia of speech mainly results from abnormalities of the superior premotor cortex. On the other hand, agrammatic aphasia presents abnormalities in the inferior frontal lobe, mainly in pars triangularis, and is also associated with abnormalities in other temporal and parietal regions [106, 107, 109, 112, 113].

Brain perfusion SPECT findings could also be useful in the estimation of the background pathology of PNFA. Nester et al. reviewed the findings of ^18^F-FDG-PET and brain perfusion SPECT on 13 pathological non-AD and 6 pathological AD patients with PNFA. Bilateral reduced accumulation of temporoparietal association cortex was observed only in three patients with pathological AD and was considered specific to pathological AD. Only 5 patients with pathological non-AD showed normal bilateral temporoparietal accumulation, which was considered specific to pathological non-AD. Although unilateral decreased temporoparietal accumulation was seen in both groups, bilateral decreased accumulation appeared in only one case of pathological AD among four patients subsequently followed up for SPECT [114].

## Miscellaneous

Idiopathic normal pressure hydrocephalus (iNPH) and prion diseases often present with dementia and can be evaluated via brain perfusion SPECT, with both showing characteristic findings. Additionally, vascular dementia can present with various perfusion patterns corresponding to cerebrovascular disease, and remote effects can be observed. Vascular dementia can coexist with other pathologies, making it essential to evaluate for hypoperfusion that suggests other pathologies, such as AD [7, 115].

### Brain perfusion SPECT findings in iNPH

**Fig. 10:**
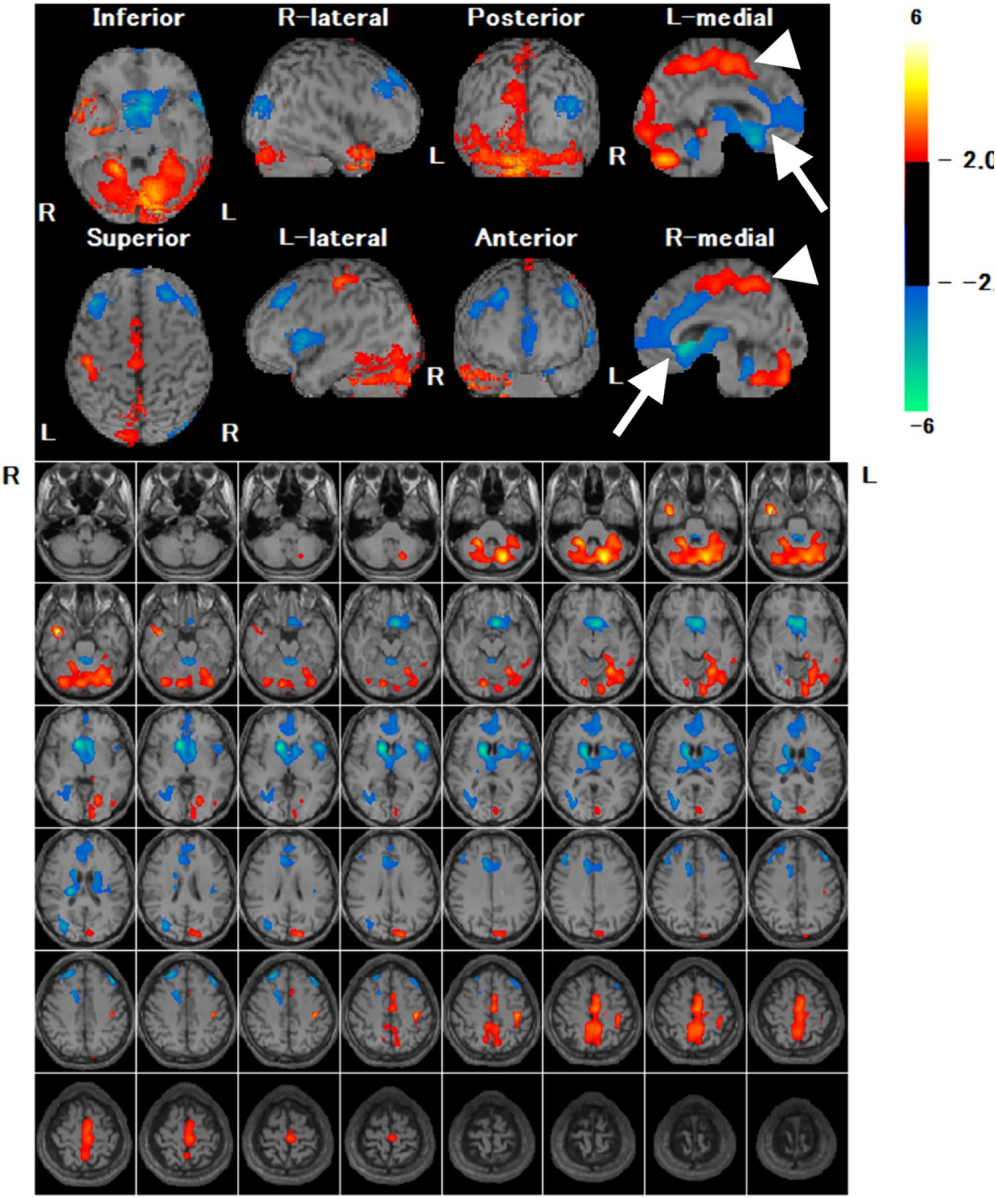
A representative case of iNPH. eZIS analysis of ECD brain perfusion SPECT images showed CAPPAH sign (arrowheads) and apparent hypoperfusion around the ventricles (arrows)

iNPH, which occurs without secondary causes of NPH, is characterized by the clinical triad consisting of gait disturbance, cognitive decline, and urinary incontinence in patients with ventricular enlargement and a normal mean intracranial pressure. Tight high convexity and enlargement of the Sylvian fissure, collectively called disproportionately enlarged subarachnoid space hydrocephalus (DESH), are important for diagnosis of iNPH [116]. Brain perfusion SPECT revealed hypoperfusion around the Sylvian fissure and corpus callosum, attributed to the enlargement of the Sylvian fissure and ventricles [117]. Conversely, high-convexity areas showed hyperperfusion. This finding is called the convexity apparent hyperperfusion (CAPPAH) sign (Fig. 10), which is assumed to reflect an increase in gray matter density due to tight high convexity rather than an actual increase in perfusion [116]. The term “CAPPAH” was inspired by the “kappa,” which refers to a water imp from Japanese folklore depicted as wearing a plate of water on its head. It has been reported that iNPH patients without CAPPAH sign tend to have a worse cognitive decline and a lower rate of improvement after the tap test than those with CAPPAH sign, suggesting that the CAPPAH sign may be useful in the prediction of treatment response [116]. It should be noted that apparent perfusion changes reflecting DESH do not necessarily correlate with the pathophysiology of dementia, since DESH can also manifest in asymptomatic elderly patients [118]

### Brain perfusion SPECT findings in prion diseases

**Fig. 11:**
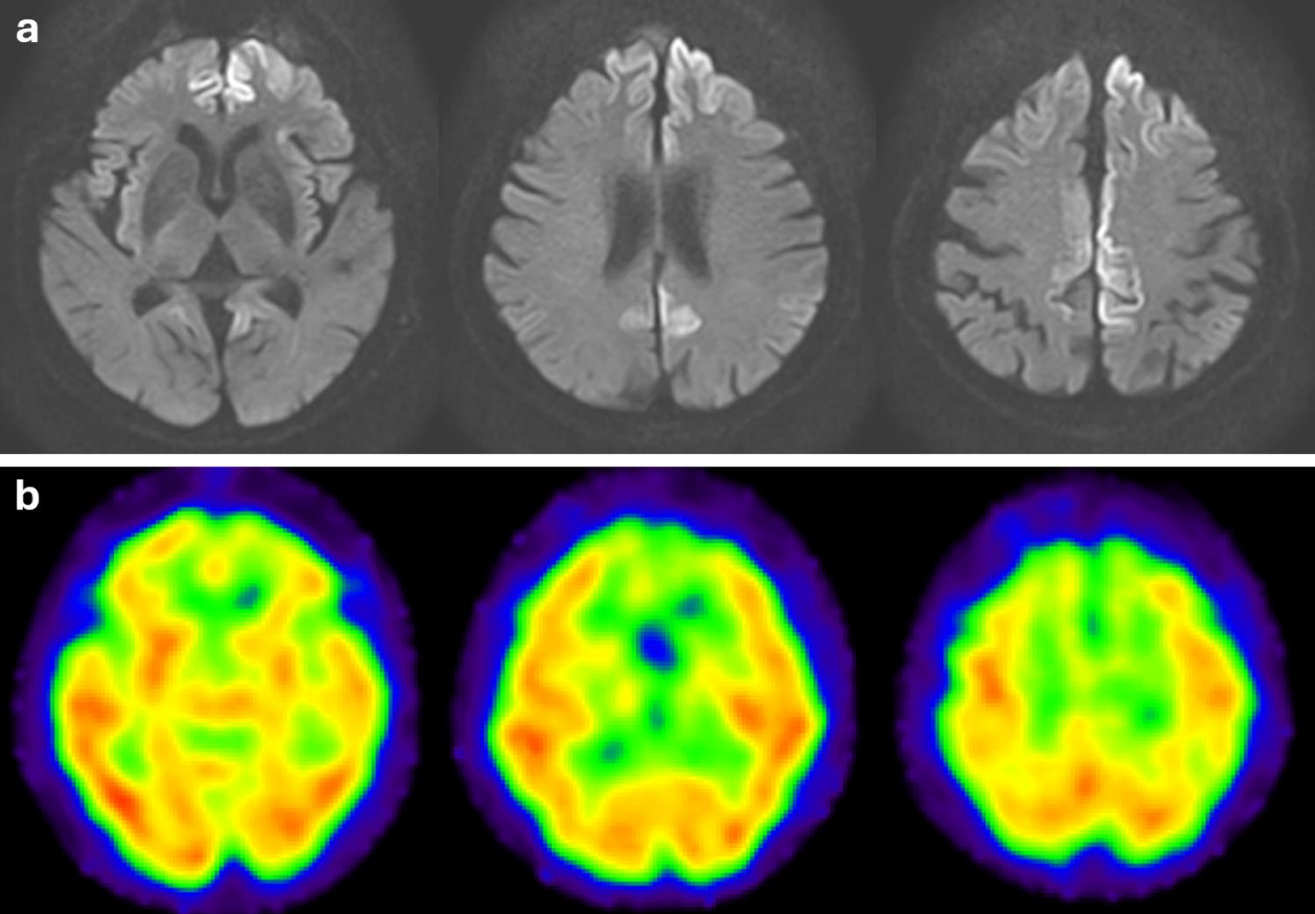
An 87-year-old male with progressive cognitive impairment diagnosed with Creutzfeldt–Jakob disease. **a** Axial DWI showed a high-intensity area along the cortex, and **b** IMP brain perfusion images revealed hypoperfusion in the same area

Prion diseases are rare, lethal neurodegenerative diseases caused by misfolding of prion proteins [119]. They are classified into sporadic, genetic, and acquired forms with sporadic Creutzfeldt–Jakob disease being the most common [120]. Typical findings on MRI include a high signal intensity on diffusion-weighted imaging (DWI) and low apparent diffusion coefficient values in the cortex and striatum [121]. This abnormal signal reflects spongiform degeneration with neuronal loss [122, 123], and brain perfusion SPECT shows hypoperfusion in the same area (Fig. 11) [124–126].

## Conclusion

Brain perfusion SPECT reveals findings that mainly reflect neurodegeneration and contributes to dementia evaluation. In this review, we summarized the knowledge on representative diseases such as AD, DLB, and FTD, and discussed iNPH and prion diseases as diseases that present with dementia and have characteristic brain perfusion SPECT findings. While the advent of disease-modifying therapy for AD has made more accurate diagnosis of dementia necessary, there are many mixed pathologies in actual clinical practice, and accurate diagnosis is not a straightforward process. However, increasing knowledge on autopsy cases has demonstrated that brain perfusion SPECT can also provide insights into background pathology. Based on accumulated knowledge, it is crucial to interpret brain perfusion SPECT images with careful consideration of the underlying pathology.
